# A Strategic Interaction Model of Punishment Favoring Contagion of Honest Behavior

**DOI:** 10.1371/journal.pone.0087471

**Published:** 2014-01-28

**Authors:** Marcel Cremene, D. Dumitrescu, Ligia Cremene

**Affiliations:** 1 Technical University of Cluj-Napoca, Cluj-Napoca, Romania; 2 Babes-Bolyai University of Cluj-Napoca, Cluj-Napoca, Romania; University of Maribor, Slovenia

## Abstract

The punishment effect on social behavior is analyzed within the strategic interaction framework of Cellular Automata and computational Evolutionary Game Theory. A new game, called Social Honesty (SH), is proposed. The SH game is analyzed in spatial configurations. Probabilistic punishment is used as a dishonesty deterrence mechanism. In order to capture the intrinsic uncertainty of social environments, payoffs are described as random variables. New dynamics, with a new relation between punishment probability and punishment severity, are revealed. Punishment probability proves to be more important than punishment severity in guiding convergence towards honesty as predominant behavior. This result is confirmed by empirical evidence and reported experiments. Critical values and transition intervals for punishment probability and severity are identified and analyzed. Clusters of honest or dishonest players emerge spontaneously from the very first rounds of interaction and are determinant for the future dynamics and outcomes.

## Introduction

Dynamics of honest/dishonest behavior in social and economic environments are of particular interest as they are important influencers of social functionality. Honesty and trust are considered the main foundations of social order. Dishonesty often leads to undesired social phenomena undermining common welfare [1]. Erosion of social norms deteriorates the social capital [2]. Moreover, in the economic realm, dishonest behavior and the associated corruption may generate market failure and poverty [3].

The 2008 crisis illustrates the effects of dishonest behavior. Toxic and risky financial products have been used only because they offered huge profits for banks and investment funds. The lack of regulation and control made this possible. When the tailors of such speculative schemes are questioned about their deeds the answer is most often: ‘because we can, because the control is loose, because state authorities lack the resources to catch people like me’ (see the declarations of Bernard Madoff - operator of the largest financial fraud in U.S. history). It has been proven that people often cheat when the punishment certainty is low and also because other people do it [4]. Nowadays, due to the wide spread of information technology, the contagion of such behaviors is easier and faster than before [5].

Dishonesty, defined in the context of personal interactions, may be seen as a strategy that increases the benefit of the one using it while causing loss to the other interacting person. The principle is: ‘one man’s bribe may be another man's gift' [6]. In other words, dishonesty is a socially defecting choice. An individual may have an incentive to act dishonestly in order to increase his/her payoff (i.e. material or of any kind). This usually leads to lower payoffs for those interacting with him/her. Advantages and also risks are associated to acting dishonestly. The benefit may be seen as the payoff of crime [7], whereas the costs include material costs, status risks, the probability of being caught, and the prospect of penalty.

A typical scenario where social honesty dynamics may be observed is based in a society where each player (individual, group, firm, etc.) interacts with other players (providing, for instance, services, products, information). Every interaction is actually a transaction. Moreover, an explicit or non-explicit competition is the underlying mechanism. Players may establish an informal (possibly verbal) contract for providing/using a certain service. An honest behavior consists, for instance, in offering a service/product at the expected quality, whereas a dishonest behavior means cheating by deliberately providing lower quality, misleading or incomplete information, fake results, or by causing delays.

The existence of some kind of punishment of dishonest behavior seems to be a necessary condition for promoting, supporting, encouraging, and protecting honesty [8]–[10]. Various natural or spontaneous forms of punishment exist [11] - be it a penalty or just a critical feedback from another player. A punishment may be applied by a player, a group of players or by a central authority.

Altruistic punishment, for instance, is one of the mechanisms that may enforce cooperation in human societies [12]. Punishment is called ‘altruistic’ if it implies a cost for the punisher and if the punished person's behavior is meant to change for the benefit of the society. An example is to tell a queue jumper to stand in line. The negative emotion produced by the defector is a possible mechanism for triggering an altruistic punishment. Experiments with humans confirm this hypothesis [12].

An important issue about punishment is to find an efficient balance between severity and certainty [13]. Empirical evidence and reported experiments reveal that punishment certainty is more important than severity [14]. Moreover, social experience indicates that a low punishment probability is inefficient even when punishment severity is very high [11]. A classical example is the U.S. Prohibition.

The general subjective perception about the risk of being punished is also important. Dishonest behavior is encouraged when the perceived risk of punishment is low [15]. In societies affected by deep systemic corruption, it is very difficult to eradicate dishonest behaviors because bad habits are somehow culturally accepted and considered more or less ‘normal’.

Both honest and dishonest behavior may display an *epidemic character* [16]. A successful yet dishonest person may be taken as a model by others. The notoriety of ‘successful’ negative models may increase their negative impact by replication of their actions by other individuals. An individual's incentive to unethical behavior depends largely on social norms. The important figures in their social group will have a larger impact [16].

Social conformism, need for safety, greed, and the fear of missing out induce powerful incentives for imitation [5]. Imitation becomes a convenient heuristic when there is too much information to process [5]. Also, imitation is an effective mechanism of spreading a social behavior. Then *How to start and promote an honesty epidemic?*

We study the conditions that favor (or not) the emergence of honest behavior in a social framework. Punishment, in its various forms, penalty or negative feedback, is considered a control mechanism. The main control parameters are the certainty and the severity of punishment - which eventually can act as honesty contagion incentives. We posit that an appropriate/finely tuned punishment mechanism may start an epidemic of honesty. We assume that community members and/or an authority are able to discover and punish, with a certain probability, dishonest players. Zero punishment probability may account for a corrupt/dishonest-dominated society. However, public policies may finely tune some parameters that influence players' payoffs, in various situations.

Honest/dishonest behavior dynamics under punishment effect are analyzed in the strategic interaction framework of Cellular Automata and Evolutionary Game Theory (GT). Strategic interactions are the basic paradigm of GT: one player's payoff depends on the actions of all the other players in the environment. We propose a social dilemma game, called the Social Honesty (SH) game. Players' strategies are either ‘honest’ (H) or ‘dishonest’ (D). We study the influence of local social interactions on the spreading of a particular behavior. Numerical simulations explore the contagion dynamics of honest and dishonest strategies in the population.

### Approach

Internal mechanisms that trigger human behavior are too complex to be accurately described and to be captured by sound (mathematical or computational) models. Social interactions add even more complexity. That is why a feasible approach would be that of focusing on the individual behavior, as it is relatively easy to observe and measure [17]. Accordingly, honest and dishonest actions may be seen as classes of human behavior/action.

Becker proposes a simple economic model: a rational crime theory [7]. According to this model, an individual decides to commit a crime if the revenue obtained is higher than the price payed for that crime. This model was proven to be inadequate for many real world situations [4], [16]. This is due to the fact that human behavior is significantly influenced by non-material aspects such as: emotions and beliefs, the perceived risk of punishment, the salience of ethicality, the visibility of unethicality of another person, social identity, reputation, reciprocity, etc.

Honest/dishonest behavior translates into cooperation/defection in social dilemma games. Emergence of social cooperation has been extensively studied in the framework of public goods games - tragedy of the commons, prisoner's dilemma, collective action logic, etc. [18]–[24]. Also, an important number of publications related to the evolution of cooperation takes a combined approach interleaving concepts from Game Theory and Evolutionary Theory. Several evolutionary game models proved successful in explaining biological phenomena and human behavior (i.e. [23]–[29], etc.).

We base our approach in the fields of Cellular Automata and Evolutionary Game Theory. We propose a new social dilemma game called the Social Honesty (SH) game. In order to capture the uncertainty of the social environment the SH game model considers probabilistic payoffs. Payoffs are described as random variables.

For convenience, we call an ‘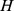-player’ a player using the honest strategy and a ‘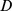-player’ a player using the dishonest strategy. We assume that an incentive towards dishonest behavior exists, yet there is also an associated risk: a probabilistic punishment for 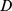-players.

When both players chose the dishonest strategy only one of them will win, yet the punishment may be applied to both of them. Dishonest behavior in one player causes a lower payoff for the honest player with whom he/she interacts.

Table 1 depicts the payoff matrix of the Social Honesty game.

**Table 1:** SH game normal-form.

Player1/Player2	Honest ( 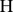 )	Dishonest ( 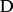 )
Honest (*H*)	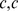	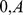
Dishonest (*D*)	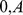	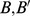

The payoff matrix of the Social Honesty game. Two-player normal-form game.

Within the SH game, when two 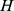-players interact each player gets a positive payoff 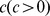. The value of 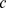 is constant.

When an 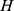-player interacts with a 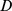-player, the 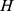-player gets zero and the 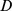-player is punished with probability 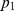.

Let us denote by 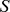 be the punishment severity (usually 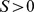). If not punished, the 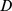-player gets a payoff equal to 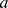, which represents the 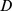-player's advantage in an 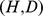 interaction (advantage to be dishonest). Thus, 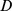-player's payoff may be expressed as a discrete random variable 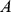. Variable 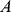 takes the value 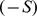 with probability 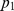 and the value 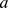 with probability 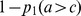. 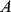 is defined as follows: 
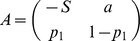



In (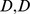) interactions, payoffs are assigned to players according to the following rule:

(*i* ) each 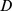-player may be punished independently with probability 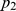;
(*ii*) if no player is punished, one player gets zero and the other one gets 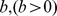, or the opposite, with equal probabilities.

Therefore 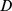-players cannot win 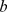 and zero simultaneously, but both may be punished.

The payoffs for the 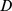-players may be expressed as discrete random variables 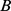 and 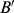, defined as follows: 
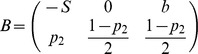
 and 
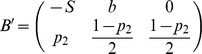



#### Spatial form of SH game

A standard 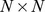 lattice model is considered. Each lattice cell represents a player. In the majority of our experiments players are arranged on a regular lattice with joint boundaries of a *cellular automaton* [30], [31] (see also [32], [33]). Another set of experiments are based on scale-free networks [34].

The state of a cell is the strategy of the corresponding player (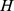 or 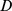). At each game round a player may act either honestly or dishonestly. Each player strategically interacts with the neighbors by means of the SH game. A player's gain in one round is the sum of the payoffs obtained in each of her interactions of that particular round. Players synchronously update their strategies at each round.

Experiments are based on Moore, von Neumann [35], well-mixed and scale-free neighborhood topologies [34]. In most of the subsequent experiments we use Moore's neighborhood with radius 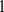 (eight cells surrounding a central cell). One experiment is dedicated to a comparison between different types of neighborhoods.

Human learning is a complex process based on social and asocial forms of learning [36], [37]. Our aim is not to find the best suited form of learning but to test the most important learning strategies and compare them. Therefore, several strategy update rules are experimented. ‘Best’ imitation strategy is used in the majority of the experiments: each player imitates the strategy of the neighbor with the highest payoff of the last round. This strategy update rule, inspired from the ‘survival of the fittest’ principle, is frequently used in evolutionary games [38]. Probabilistic strategy update rules, based on myopic and Fermi function [39], [40], are also experimented.

Experiments run on the following parameter setting of the SH game: 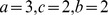. In most of the subsequent experiments we use a punishment probability of 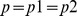. In one experiment we use unequal punishments. Experiments are performed on a 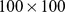 population. For statistical relevance, results are averaged over 100 runs.

## Results

The main findings of our experiments are described in the following:

### Experiment 1. Emergence of 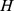 and 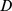 clusters in the population

In this experiment we start from an initial 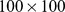 population with 50% 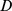-players, randomly distributed. Punishment severity is 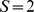 and punishment probability is 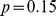. Radius 1 Moore neighborhood and best-neighbor imitation update strategy are used.

**Figure 1:**
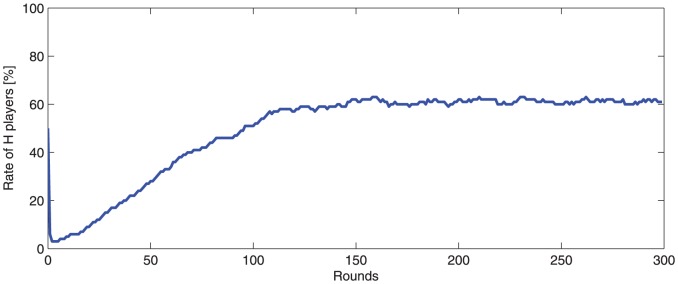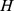-player rate dynamic in a 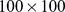 population in the first 300 rounds (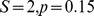). The initial population contains 50%, randomly distributed, 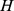-players. The 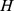 rate drops dramatically in the first three rounds, to about 2%. 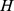-player rate constantly increases in the next rounds. After 20 rounds the 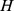-player rate is about 8%. After 50 rounds there are 24% 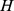-players. After 300 rounds the 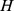-player rate is about 60% and remains almost constant indicating that an equilibrium is reached.

**Figure 2:**
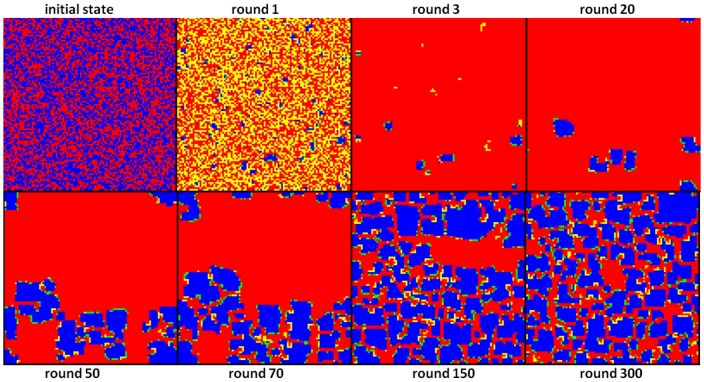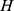-cluster formation in a 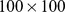 population after 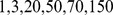, and 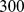 rounds (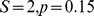). The initial population contains 50%, randomly distributed, 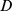-players. Few small clusters of 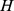-players appear in the very first rounds, containing only 2% 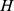-players after round 3. After 50 rounds the 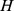-clusters become larger (25% 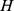-players). After 150 rounds 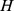-clusters may be found all over the population (57% 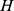-players). The color code is: blue - is honest/was honest; red - is dishonest/was dishonest; green - is honest/was dishonest; yellow - is dishonest/was honest.

Fig. 1 and Fig. 2 illustrate honest/dishonest the population dynamics over the first 300 rounds.

It may be observed that the 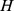-player rate drops dramatically in the first 3 rounds. Only few small 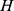 clusters survive. In time, these clusters grow and divide. Cluster shape and location are changing dynamically. 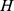 and 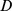-player rates become approximately stable after about 150 rounds and remain almost unchanged for 100,000 rounds (60% 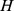-players, 40% 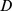 players), indicating a kind of dynamic equilibrium.

As it may be observed in Fig. 2, 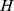-player cluster formation seems to be an important phenomenon in resisting 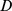-player invasion. Cluster dynamics indicate that numerous changes occur at the cluster frontiers, and few or no changes in the cluster center.

**Figure 3:**
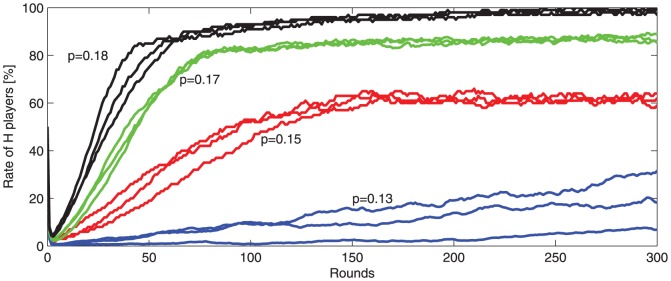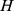-player rate dynamics in the 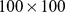 population, first 300 rounds. Different game runs are depicted, corresponding to different punishment probabilities: 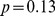 (3 runs, blue), 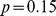 (3 runs, red), 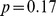 (3 runs, green), and 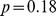 (3 runs, black). 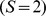. The initial population contains 50% 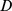-players, randomly distributed.

Player rates at equilibrium (approximately constant rates) depend on the punishment probability and severity. Fig. 3 depicts the honest/dishonest dynamics in the first 300 game rounds for different punishment probabilities (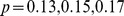 and 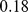) and constant punishment severity (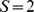). In each case, the 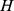-player rate drops very fast and then increases slowly, remaining quasi stable after 300 rounds.

**Figure 4:**
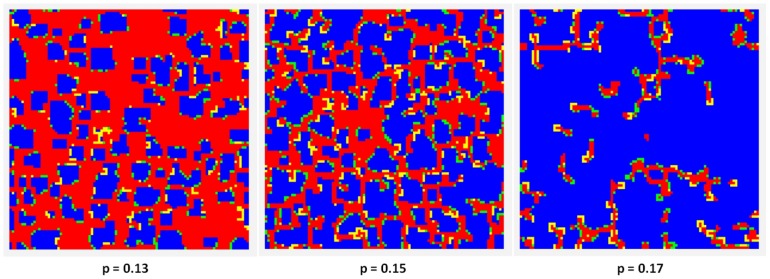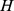-cluster formation in a 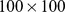 population after 1000 rounds, for different punishment probabilities 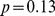, 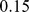, and 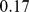. 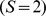. The initial population contains 50%, randomly distributed, 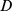-players. The color code is: blue - is honest/was honest; red - is dishonest/was dishonest; green - is honest/was dishonest; yellow - is dishonest/was honest.

The cluster pattern depends on parameters 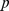 and 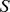. Fig. 4 depicts the population state after 1000 rounds for 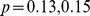, and 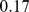 (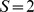).

A dynamic equilibrium establishes in time. The rate of honest and dishonest players in a population depends on the values of punishment probability 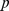 and punishment severity 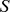 and remains constant after a number of rounds if 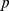 and 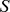 are kept constant.

### Experiment 2. Punishment impact on 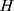 and 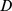-player rates in the population

In this experiment the player rates are measured after 500 rounds. Each experiment runs for 100 times and the results are averaged. We start from an initial 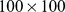 population with 50% 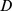-players, randomly distributed. Radius 1 Moore neighborhood and best-neighbor imitation update strategy are used.

**Figure 5:**
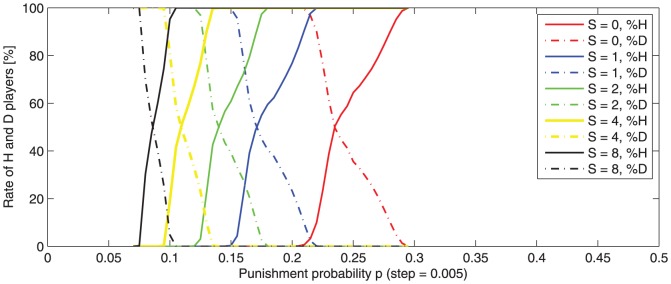
Average variation of 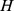 and 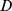-player rate with punishment probability 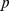. Averaged values for 100 runs are observed after 500 game rounds. The game starts with 50%, randomly distributed, 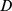-players. Punishment severity varies: 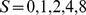. For lower 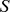, 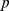-transition intervals become wider and translated to higher values. The 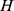 rate becomes 100% if the punishment probability 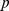 is higher than a specific value: 0.295 for 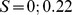 for 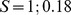 for 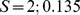 for 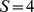, and 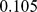 for 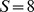.

Fig. 5 depicts the rate of 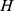 and 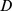-players for different punishment probabilities (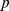).

Some 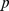-transition intervals are identified, accounting for a translation from an average 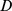 domination to an average 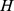 domination (Fig. 5). In order to guarantee 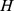-player domination, punishment probability should be higher than the upper bound of the 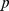-transition interval.

Table 2 illustrates the 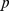-transition intervals for different values of the punishment severity 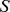.

**Table 2:** Experimental 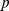-transition intervals.

Punishment severity 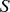	Experimental 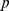 -transition interval
0	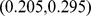
1	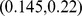
2	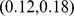
4	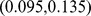
8	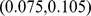

Experimental 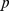-transition intervals 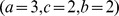 for 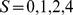, and 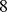. The game starts with 50% 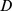-players randomly distributed. Average 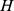-player rate values for 100 runs are observed after 500 game rounds.

**Figure 6:**
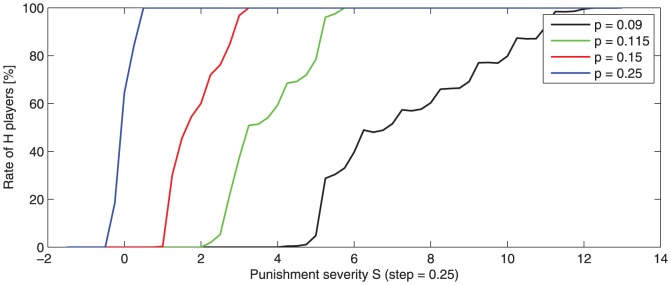
Variation of 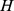-player rate with the punishment severity 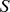. Averaged values for 100 runs are observed after 500 game rounds. Punishment probability 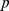 is: 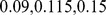, and 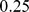. The game starts with 50%, randomly distributed, 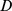-players.

Similar to 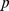-transition intervals, specific transition intervals for the punishment severity have been found. 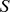-transition intervals are depicted in Fig. 6.

For lower 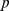 values, the 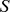-transition intervals become significantly wider and translated to higher values. This means that punishment severity is ineffective when punishment probability is very low. Higher punishment probability makes it possible to reduce significantly punishment severity, with the same effect on the 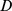 and 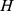 population rate.

### Experiment 3. The effect of increasing the advantage of being dishonest

If 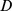-player's advantage when playing against an 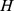-player is double (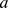 is set to 6 instead of 3) the effect is a significant change in the 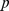-transition interval: from 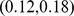 to 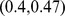, indicating a large increase in the required punishment probability. This indicates that 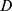-player's advantage in a 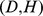 interaction is a sensitive parameter of the model.

### Experiment 4. The effect of unequal punishment probabilities on the 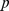-transition interval

If 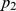 is set to 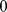, meaning that a 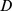-player is punished only when playing against an 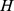-player, the effect is a translation of the 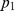-transition interval, which also becomes slightly narrower. For 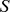 = 2 and 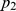 = 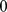, the 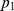-transition interval changes from 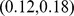 to 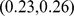. This indicates that a double punishment probability is necessary when, for some reasons, 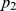 is close to zero. This corresponds to a case when both 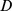-players are difficult to expose. Such a form of cooperation between 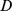-players is clearly unfavorable to 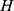-player spreading.

For the rest of experiments we set 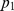 = 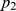 = 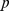.

### Experiment 5. The effect of the initial rate of 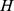 and 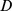-players on the 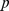-transition interval

**Figure 7:**
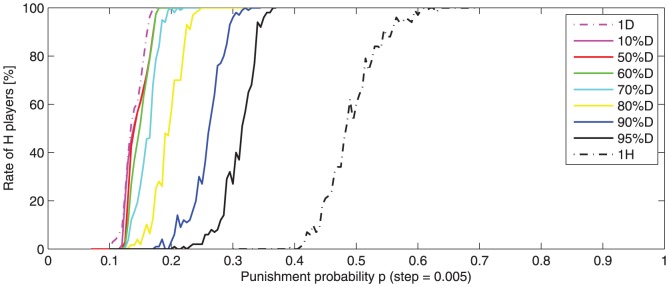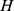-player averaged rate (100 runs) after 500 rounds, function of punishment probability 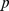, (for 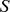 = 2). Different initial states are considered: one 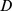-player in the middle, 10%, 50%, 60%, 70%, 80%, 90%, 95% 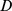-players randomly positioned and one 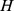-player in the middle. In all cases, a 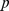-transition interval from 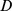 dominance to 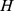 dominance appears. The interval is wider and translated to higher values for higher initial rates of 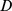-players. Significant changes appear only for more than 60% 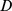-players.

Different rates of randomly distributed 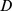 and 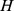-players in the initial population are considered. Fig. 7 illustrates the effect of the initial 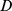-player rate on the 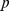-transition interval.

The 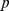-transition interval changes when the initial rate of 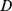-players changes. The difference is significant for a 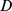 rate superior to 50% (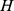 rate below 50%) and less significant when 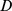-players are a minority. Between one 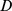-player and 50% 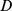-players the 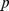-transition interval does not change much. When 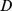-player rate is high (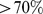) the 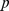-transition interval becomes more nosy.

An explanation may be found if we correlate these results with the observations about cluster dynamics (see Experiment 1, Fig. 1 and 2). When the initial rate of 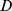-players is high, the 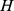 cluster formation probability is low. If no 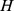 cluster appears in the first rounds, then 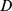-players will spread all over the population.

This suggests the fact that the initial cluster structure is more important than the initial proportion of 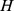 and 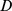-players. The importance of the initial cluster structure is investigated in the next experiment.

### Experiment 6. The importance of the initial cluster formation

In this experiment we start from a situation where clusters of 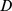 and 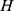-players already exist (in all other experiments we start from 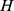 and 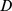-players randomly spread).

Two world states are generated by letting the population evolve: one with 95% and the second with 12% 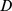-players. In both cases clusters already exist (similar to what is seen in Fig. 4). The granularity is measured by counting the strategy changes for each lattice row. The value is averaged and normalized. The granularity is similar for the two cases (about 0.9, whereas in the case of randomly spread players it is about 0.5).

**Figure 8:**
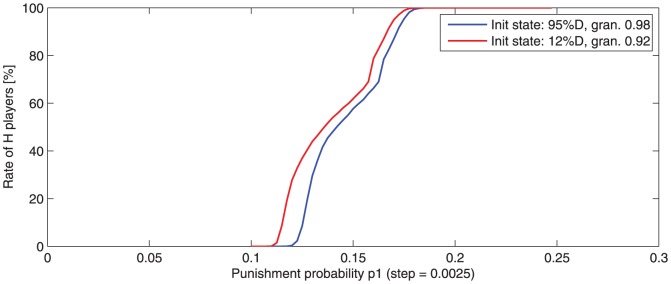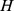-player averaged rate (100 runs) after 500 rounds, function of punishment probability 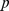, for 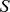 = 2. Two initial states are considered: one with 95% and the second with 12% 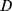-players, both containing already formed clusters of players. The 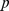-transition intervals are very similar, despite the initial player rate. A granularity measure (0 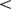 gran. 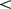 1) is used for characterizing the clusters (a high value indicates few large clusters).

The results are depicted in Fig. 8.

It may be observed that 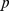-transition intervals are very similar for the two different initial world states. This fact indicates that cluster existence is much more important than the initial 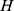/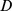 population rate.

### Experiment 7. The effect of the population size on the 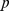-transition interval

**Figure 9:**
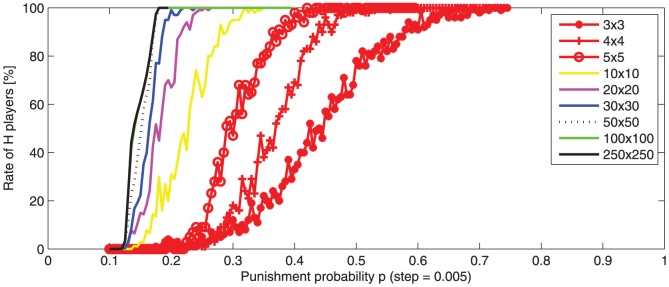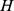-player averaged rate (100 runs) after 500 rounds, function of punishment probability 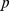, (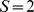). The initial population contains 50%, randomly distributed, 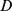-players. The 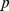-transition intervals are depicted for different population sizes (from 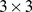 to 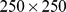). For small populations, 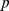-transition intervals are significantly wider and translated to higher values. A noise, caused by a high dispersion of the 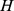 rate dynamic, appears for small populations.

In this experiment we study the effect of the population size. Fig. 9 depicts the average 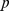-transition intervals for different population sizes: 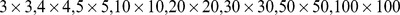, and 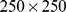.

It may be observed that, for small size worlds, the 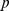-transition intervals are wider, translated to higher values, and also exhibit some noise. Significant changes appear when the world size is smaller than 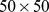.

Since the values depicted in Fig. 9 are averages, the noise indicates a high dispersion of the 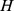 rate dynamic. The noise observed for the small size worlds indicate that their dynamic is less stable. These results may be explained by the initial cluster formation: in a small size world, the cluster formation probability is lower than in a large world. If 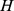 clusters do not appear, the situation converges rapidly to a pure 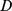 dominance.

A similarity may be observed between the 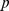-transition interval for a small population (Fig. 9) and the 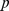-transition interval for an initial world state with numerous 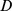-players (Fig. 8). This similarity may be explained by a common cause: the probability of 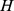 cluster formation in the first rounds depends on the initial distribution but also on the population size (probability of cluster formation is higher in larger populations).

We notice that higher punishment probability and severity are needed in small-size worlds (e.g. 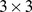 or 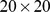) in order to obtain the same effect as in larger size worlds (e.g. 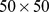 or 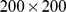).

**Figure 10:**
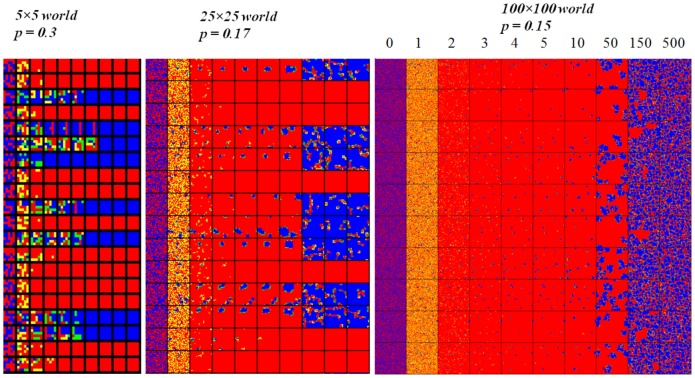
Different-size world dynamics: a 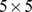 world, a 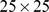 world, and 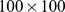 world. Several simulation runs (each on a separate row are depicted. The initial population contains 50% 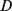-players randomly distributed. 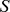 = 2 and 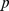 is selected in the middle of the 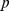-transition interval for each 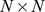 world. 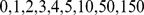, and 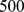 rounds of the SH game are captured. The color code is: blue - honest/was honest; red - dishonest/was dishonest; green - honest/was dishonest; yellow - dishonest/was honest.

As we already noticed, cluster formation is the main driver for spreading honest/dishonest behavior. Fig. 10 depicts three different-size world dynamics.

Very small size worlds tend to converge towards a pure distribution (100% 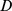 or 100% 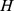). In medium and large size worlds dynamic equilibria of mixed populations appear when 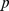 or 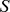 are within the transition intervals.

### Experiment 8. The effect of the neighborhood type on the 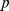-transition interval

In this experiment we analyze the impact of different types of neighborhoods: von Neumann, Moore, well-mixed, and scale-free. Von Neumann and Moore neighborhoods may have different radia. In a ‘well-mixed’ case everybody is neighbor with everybody. In a ‘scale-free’ neighborhood the connections are no longer related to the original lattice structure. Instead, a spatial power-law based graph is mapped on the lattice (each lattice node is a vertex in the scale-free graph).

**Figure 11:**
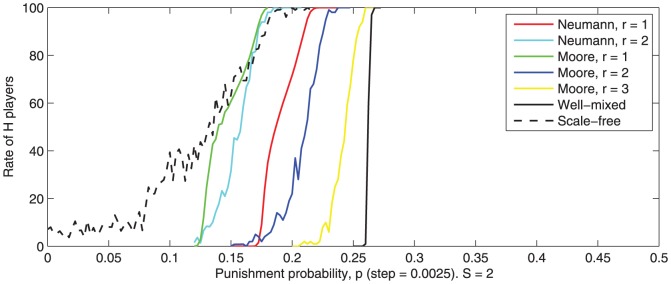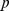-transition intervals for different types of neighborhoods. von Neumann (r = 1,2), Moore (r = 1,2,3) well-mixed (everybody is neighbor with everybody) and scale-free (power law connections mapped on the lattice). Averaged values for 100 runs are observed after 500 game rounds.

Results are depicted in Fig. 11.

As expected, the 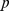-transition interval is influenced by the neighborhood type. However, this influence is rather minor and a phase transition appears in all cases. In the ‘well-mixed’ neighborhood network, the 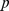-transition interval is narrower and translated to higher values.

For the ‘scale-free’ topology, the 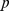-transition interval is wider. The upper bound of this interval is close to the upper bounds of the intervals obtained for von Neumann and Moore neighborhoods. For low punishment probability, the; ‘scale-free’ topology is more favorable to 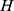-players.

### Experiment 9. The effect of the strategy update rule on the 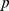-transition interval

In this experiment we analyze the 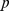-transition intervals for different types of strategy update rules. When using the ‘Best’ rule, players imitate the best player (i.e. the neighbor with the highest payoff). With ‘Best Myopic’ players imitate the best player with probability 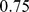 and a randomly chosen neighbor with probability 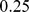. With ‘Best Fermi’ the best player is imitated with a probability given by a particular function proposed in [40] or keeps its strategy unchanged.

**Figure 12:**
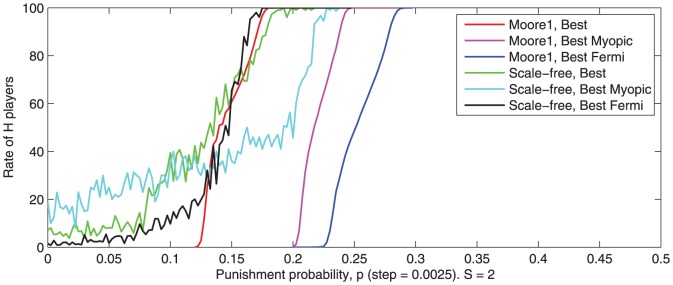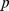-transition intervals for different types of strategy update rules. ‘Best’ rule - players imitate the best player (i.e. the neighbor with the highest payoff). ‘Best Myopic’ rule - players imitate the best player with probability 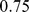 and a randomly chosen neighbor with probability 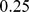. ‘Best Fermi’ rule - the best player is imitated with a probability given by a particular form of Fermi function. Averaged values for 100 runs are observed after 500 game rounds.

Results are depicted in Fig. 12.

The strategy update rule influences the 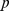-transition interval by slightly changing its position. When using Moore neighborhood and ‘Best Fermi’ update rule a higher punishment probability is required in order to obtain the same effect as when using the ‘Best’ strategy. When using a ‘scale-free’ topology the difference between ‘Best’ and ‘Best Fermi’ rules is less significant.

## Conclusion

A social dilemma model, called the Social Honesty (SH) game, is proposed and investigated. The SH game induces complex dynamics when iterated on a regular grid (cellular automaton) or network of various topologies and using different strategy update rules. The emerged dynamics may offer relevant insights onto real world processes such as social behavior dynamics.

Experimental results illustrate how the behavior of a population of interacting individuals may be influenced by setting an adequate punishment severity and applying it with a certain probability, even up to the point where an honesty domination may be achieved. Experiments indicate that punishment probability is more important than punishment severity. These results confirm the empirical evidence and studies based on real world observations [13], [14], thus illustrating the validity of the model.

Transition intervals for punishment probability and severity have been identified. The results indicate the presence of transition intervals in all experiments. Punishment severity proves to be ineffective when punishment probability is very low. Higher punishment probability makes it possible to reduce significantly the punishment severity, with the same effect on the honest/dishonest population rate. These results may be related to the observations about the U.S. Prohibition period when, despite a high punishment severity, the punishment probability was very low and thus ineffective [11].

When a ‘zero tolerance’ policy [41] is too costly and difficult to implement in practice, a solution is to find the punishment transition interval in order to finely tune the control mechanism. The optimal combination between punishment severity and probability depends on the involved costs.

Also, a higher punishment probability proves necessary when the dishonest's advantage is higher. Apparently, this result, confirming the rational crime model [7], may contradict the conclusions of [4], where the amount of money and punishment probability do not influence the temptation to act dishonestly. However, the considerations from [4] concern one individual, whereas in our model the individuals are influenced by their neighbors, through imitation.

Another finding is that the honest strategy survival depends on the players' ability to form clusters. A similar phenomenon has been observed for cooperative behavior emergence in experiments with Prisoner's Dilemma game [32], [33]. We find that initial proportion of 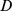 and 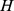-players is less important than the initial cluster formation (groups are stronger against aggression than isolated individuals). Also, small size populations seem to be less predictable and less sensitive to punishment.

Results indicate that the proposed model may describe real world phenomena with an acceptable approximation. New dynamics, with a new relation between punishment probability and punishment severity, are revealed. An epidemic of honesty is possible if model parameters are finely tuned and the cluster formation is triggered. Hopefully, policy makers, various groups and organizations, and even law enforcement institutions may use such a model for fine tuning punishment severity and certainty towards favoring honest behavior contagion.

As future work we intend to enrich our model with some additional features for the player model (identity, memory, etc.), test some other network topologies and new strategy updating rules. Another direction of interest is related to real world validation experiments.
